# Supplementary material for: Feasibility Study of a Menstrual Hygiene Management Intervention for People with Intellectual Impairments and Their Carers in Nepal
Source: Int J Environ Res Public Health. 2019 Oct 4;16(19):3750. doi: 10.3390/ijerph16193750 (PMC6801804; doi:10.3390/ijerph16193750)
Supplement: Supplementary file 1 [file ijerph-16-03750-s001.zip › S1_feasibility_study_indicators_and_results.docx]

**Table S1**. Feasibility study indicators and results

| No. | Indicator | Achieved (Y/N) | Comments |
| --- | --- | --- | --- |
| 1 | 50% of young people and carers recognized as Bishesta households | N | To achieve Bishesta household status, households had to meet the household monitoring indicators.  100% of young people met the indicators, but only 10% (*n* = 1) of carers used the menstrual calendar, meaning 90% (*n* = 9) of households did not achieve Bishesta household status. |
| 2 | Carers reporting that they and / or the young person positively benefited from the involvement in the Bishesta programme | Y |  |
| 3 | 50% of participants practicing target behaviours | Y |  |
| 4 | 85% of participants attended three group training sessions | Y |  |
| 5 | All group training delivered as planned | N | One of the three group training sessions were not delivered by the intended number of facilitators. |
| 6 | Facilitators reporting effective delivery of the group training sessions | Y |  |
| 7 | Cost per young person is in line with similar pilot studies | ? | No ‘similar pilot studies’ exist, so benchmarking the costs per young person is impossible |
| 8 | Intervention could be delivered alongside general menstrual hygiene management interventions or existing disability programmes | Y |  |
| 9 | Desire to expand the programme into other areas of Nepal | Y |  |
