# Supplementary material for: Feasibility Study of a Menstrual Hygiene Management Intervention for People with Intellectual Impairments and Their Carers in Nepal
Source: Int J Environ Res Public Health. 2019 Oct 4;16(19):3750. doi: 10.3390/ijerph16193750 (PMC6801804; doi:10.3390/ijerph16193750)
Supplement: Supplementary file 1 [file ijerph-16-03750-s001.zip › S2_quant_results_and_analyses_to_qual_results.docx]

**Table S2.** Structured questionnaire results and analyses of the findings against the qualitative data.

| **Question** | **Baseline** | | | **Endpoint** |  | **Analysis** |
| --- | --- | --- | --- | --- | --- | --- |
|  | Answer | **Number of participants** | **Percentage of participants** | **Number of participants** | **Percentage of participants** |  |
| The person I care for uses a menstrual product | Always | 8 | 80% | 8 | 80% | Two more young people using a menstrual product. Qualitative data indicates a greater improvement. |
|  | Sometimes | 0 | 0% | 1 | 10% |  |
|  | Never | 2 | 20% | 1 | 10% |  |
| The person I care for is able to understand when she needs to change her menstrual product | Yes | 8 | 80% | 5 | 50% | Reduction in 3 young people’s understanding of when to change a menstrual product. Not supported by the qualitative findings. |
|  | No | 1 | 10% | 4 | 40% |  |
|  | N/A | 1 | 10% | 1 | 10% |  |
| The person I care for is able to change her menstrual product independently | Yes | 5 | 50% | 6 | 60% | One more young person able to change her menstrual product independently. This supports the qualitative findings. |
|  | No | 4 | 40% | 3 | 30% |  |
|  | N/A | 1 | 10% | 1 | 10% |  |
| The person I care for needs support to change her menstrual product | Yes | 4 | 40% | 6 | 60% | Two more young people need support to change her menstrual product. These are the two young people that are now using a menstrual product (see question 1). |
|  | No | 5 | 50% | 3 | 30% |  |
|  | N/A | 1 | 10% | 1 | 10% |  |
| The person I care for has shown her menstrual pad/blood to other people | The last time she menstruated | 0 | 0% | 0 | 0% | Two young people who showed their menstrual blood in public, have not since the intervention. Qualitative data indicates a greater improvement. |
|  | In the last two months | 0 | 0% | 0 | 0% |  |
|  | In the last three months | 2 | 20% | 0 | 0% |  |
|  | Never | 8 | 80% | 10 | 100% |  |
| The person I care for takes pain relief | Always | 0 | 0% | 4 | 40% | 40% (*n* = 4) young people always taking pain relief; 10% (*n* = 1) decrease in young people never using pain relief. This supports the qualitative findings. |
|  | Sometimes | 5 | 50% | 4 | 40% |  |
|  | Never | 3 | 30% | 2 | 20% |  |
|  | N/A | 2 | 20% | 0 | 0% |  |
|  |  |  |  |  |  |  |
|  |  |  |  |  |  |  |
|  |  |  |  |  |  |  |
|  |  |  |  |  |  |  |
|  |  |  |  |  |  |  |
|  |  |  |  |  |  |  |
| **Question** | **Baseline** | | | **Endpoint** | | **Analysis** |
|  | Answer | **Number of participants** | **Percentage of participants** | **Number of participants** | **Percentage of participants** |  |
| During the first day of the person I care for menstruation, I change her pad | Less than 1 times a day | 0 | 0% | 0 | 0% | Carers changing young person’s menstrual pad more often during the first day of menstruation. This supports the qualitative findings. |
|  | 1 times a day | 2 | 20% | 2 | 20% |  |
|  | 2 times a day | 4 | 40% | 3 | 30% |  |
|  | 3 times a day | 1 | 10% | 4 | 40% |  |
|  | 4 times a day | 2 | 20% | 1 | 10% |  |
|  | More | 0 | 0% | 0 | 0% |  |
|  | N/A | 1 | 10% | 0 | 0% |  |
| The person I care for knows where to get clean menstrual pads | Yes | 3 | 30% | 10 | 100% | 70% (*n* = 7) more young people know where to get clean menstrual pads. This supports the qualitative findings. |
|  | No | 6 | 60% | 0 | 0% |  |
|  | N/A | 1 | 10% | 0 | 0% |  |
| When the person I care for has menstrual cramps, I: | Give her a hot water bottle | 0 | 0% | 1 | 10% | Increase in carers intervening when young person has menstrual cramps and providing different pain relief options. This supports the qualitative findings. |
|  | Give her hot water to drink | 0 | 0% | 9 | 90% |  |
|  | Give her pain killer tablets | 3 | 30% | 2 | 20% |  |
|  | Let her rest | 7 | 70% | 6 | 60% |  |
|  | Do nothing | 0 | 0% | 0 | 0% |  |
|  | N/A | 0 | 0% | 1 | 10% |  |
| When the person I care for is angry, sad, upset because of her menstruation, I: | Leave her alone | 3 | 30% | 0 | 0% | Increase in carers supporting young person when they feel angry, sad, upset because of her menstruation. This supports the qualitative findings.  a) 30% (*n* = 3) less leaving the young person alone;  b) 60% (*n* = 6) increase in reassurance;  c) 40% (*n* = 4) reduction in carers saying this is not applicable |
|  | Keep her at home | 0 | 0% | 0 | 0% |  |
|  | Give her a hug | 0 | 0% | 0 | 0% |  |
|  | Reassure her | 3 | 30% | 9 | 90% |  |
|  | Do nothing | 0 | 0% | 0 | 0% |  |
|  | N/A | 4 | 40% | 0 | 0% |  |
